# Service providers’ perspectives on facilitators and recommendations for improving HIV care in Manitoba, Canada

**DOI:** 10.3389/fpubh.2025.1585604

**Published:** 2025-07-09

**Authors:** Katharina Maier, Margaret Haworth-Brockman, Enrique Villacis-Alvarez, Lauren J. Mackenzie, Laurie Ireland, Ken Kasper, Yoav Keynan, Zulma Vanessa Rueda

**Affiliations:** ^1^Criminal Justice, The University of Winnipeg, Winnipeg, MB, Canada; ^2^Department of Community Health Sciences, Rady Faculty of Health Sciences, University of Manitoba, Winnipeg, MB, Canada; ^3^National Collaborating Centre for Infectious Diseases, Rady Faculty of Health Sciences, University of Manitoba, Winnipeg, MB, Canada; ^4^Department of Medical Microbiology and Infectious Diseases, Rady Faculty of Health Sciences, University of Manitoba, Winnipeg, MB, Canada; ^5^Department of Internal Medicine, Rady Faculty of Health Sciences, University of Manitoba, Winnipeg, MB, Canada; ^6^Manitoba HIV Program, Winnipeg, MB, Canada; ^7^Nine Circles Community Health Centre, Winnipeg, MB, Canada; ^8^Department of Family Medicine, Rady Faculty of Health Sciences, University of Manitoba, Winnipeg, MB, Canada; ^9^Facultad de Medicina, Universidad Pontificia Bolivariana, Medellin, Colombia

**Keywords:** HIV care, service provider, facilitators, recommendations, harm reduction, social ecological model, qualitative study

## Abstract

**Background:**

We aimed to identify facilitators and recommendations for improving HIV care in Manitoba, Canada from service providers’ perspectives.

**Methods:**

This study is a component of a participatory action research study examining the interrelatedness of houselessness, substance use and other factors on HIV care. We conducted in-depth, semi-structured interviews with 27 HIV service providers in Manitoba (Canada). Interviews were transcribed, coded, and analyzed using a thematic approach within a socio-ecological framework.

**Results:**

We identified 11 supertheme facilitators and 15 supertheme recommendations at the intrapersonal, interpersonal, socio-cultural, institutional and structural levels. For the facilitators, subthemes include non-judgmental care (intrapersonal), focus on building relationships and trust (interpersonal), collaboration with other providers (socio-cultural), safe non-stigmatizing environments (institutional), and effective policies (structural). Provider recommendations highlight the need for structural changes, with subthemes focused on policy changes, adaptations to HIV care delivery model, harm reduction strategies, and addressing gaps in social services and mental health care.

**Conclusion:**

Service providers’ behaviours and attitudes as well as organizational processes play a significant role in PLHIV engagement and retention in HIV care. Institutional and structural changes, including flexible and mobile HIV care as well as integrated HIV and harm reduction care, are critical for increasing care uptake and retention.

## Background

Advancements in treatment, medications, and care have transformed human immunodeficiency virus (HIV) from a fatal disease into a manageable and chronic condition. However, achieving viral suppression (e.g., undetectable viral load) and maintaining optimal health requires lifelong and consistent engagement in HIV care. In line with the “HIV care continuum,” engagement in care involves that patients routinely connect with HIV care providers, attend medical appointments, take antiretroviral therapy (ART), and receive other medical care as necessary (1, 2). Studies show that levels of engagement in HIV care are influenced by the quality of relationship between patient and health provider (3–6). Thus, it is critical we gain a full understanding of how HIV care providers work to facilitate access to and engagement in care for People Living with HIV (PLHIV) and what other factors providers believe are key to increasing HIV care uptake.

In Manitoba, Canada, there is a unique convergence of complex health and social circumstances, amplified since the COVID-19 pandemic, that has exacerbated people’s risk of acquiring HIV and other co-occurring sexually transmitted and blood borne infections (STBBI; e.g., Hepatitis C and syphilis) (7, 8). Indeed, more people were diagnosed with HIV in Manitoba in 2021 and 2022 than ever before (9), with an incidence nearly 3-fold that of Canada as a whole (10). Indigenous people, people who use injection drugs (mostly methamphetamine), and people experiencing houselessness, especially young females in these populations, are over-represented among new HIV diagnoses (7, 11). Among those newly diagnosed with HIV in Manitoba, people experiencing houselessness and those using injection drugs had lower levels of anti-retroviral treatment (ART) initiation and viral suppression (11). These factors create a newer, comprehensively different epidemiology and etiology for HIV in Manitoba, compared with other parts of Canada, that is not well understood. As PLHIV’s ability to access and stay engaged in care is shaped by these broader lived realities (7, 11), it is critical we understand how HIV providers seek to facilitate care amidst overlapping social issues shaping PLHIV’s health (e.g., poverty, houselessness, substance use).

Drawing from a socio-ecological model, this article presents findings from interviews with HIV service providers about their perspectives on facilitators to HIV care in Manitoba. Originally introduced by Urie Bronfenbrenner to describe how interactions and relationships between a person and different layers of environments guide human development in the 1970’s (12), the model has since been adapted by researchers such as McLeroy et al. (13) and McClarty et al. (14) in health promotion research to shift from focusing solely on individual level behaviours, with an aim to reduce “victim blaming” (13). This adapted socio-ecological model seeks to understand how the intersections of individual, institutional, social, and structural factors influence health, as many conditions shaping health access and outcomes are beyond an individual’s control (13). For example, Manitoba providers have noted there are interpersonal (e.g., systemic racism), institutional (e.g., limited hours of operation for healthcare) and policy/structural (e.g., availability of public transportation) impediments to PLHIV obtaining consistent care (15). By examining the separate and interrelated factors that prevent but also *facilitate* respectful, accessible, and relevant care, practical and appropriate recommendations for addressing HIV in the Manitoba context can emerge.

## HIV provider perspective

While providers play a significant role in facilitating HIV treatment, qualitative research on their perspectives on what is currently working well (i.e., facilitators) to engage and retain PLHIV is relatively limited (16). Facilitators to HIV care refer to factors that support engagement in care, referring to both material (e.g., adequate income for medication costs) and behavioural aspects (e.g., non-judgmental caring providers) (5). Existing studies highlight that a trusting, respectful, and long-term HIV care provider-client relationship is critical to the health of PLHIV (4). To illustrate, Mallinson et al.’s interviews with “hard to reach” PLHIV demonstrate that interactions they consider validating, where they felt welcome and accepted, and viewed themselves as partners in making care decisions together, facilitated engagement and retention in HIV care (17). Green and Wheeler’s study with aging gay men reported provider characteristics such as showing empathy, understanding, and knowledge about LGBTQ-specific health issues as enabling factors (5). Similarly, providers in Gelaude et al.’s study reported that engaging clients on a personal level and showing empathy and understanding for their lives are key facilitators to care (18). Mosack and Wendorf demonstrate how HIV providers can leverage informal (e.g., family) supports to enhance patients’ care (16). Organizational facilitators have also been shown to encourage PLHIV’s sustained engagement in care. For example, Yehia et al. demonstrated clinic services such as transportation support, appointment reminders, and an accessible scheduling process are key facilitators that supported PLHIV retention in care (3). Several studies report on the usefulness of integrating HIV services with other health services in one location (3) or collaborating with other service organizations to provide more comprehensive support to meet PLHIV’s varying and often complex needs (19). More specifically, researchers have found that integrating harm reduction services with HIV treatment for PLHIV who use substances can facilitate engagement in HIV care (20–22). Yet, HIV providers must navigate the complexities of chronically under-resourced and strained health care systems (23–25), further challenged by the COVID-19 pandemic, which can hinder efforts to provide optimal care (26–28).

Some studies have examined facilitators to HIV care specifically for PLHIV who use substances. For example, Grau et al.’s study with service providers in Southern New England found that cultural competence and a non-judgmental approach are key to building trust with PLHIV who use substances as they find they are often “among the most difficult” to retain in care due to substance-use related stigmatizing experiences in health settings (29). Collins et al., based on interviews with PLHIV who use drugs in Vancouver, Canada, call for enhanced integrated services to boost PLHIV’s access to health *and* social services (30). Campbell et al. examine how health providers may assist PLHIV who use substances to increase ART adherence, such as by motivating medication adherence, helping patients with scheduling when to take their medications, and building solid patient rapport (31). These studies highlight that PLHIV who use substances often face added barriers to accessing and engaging in HIV care. Thus, it is important to further examine how service providers seek to facilitate care uptake, particularly for people with intersecting health and social issues, as is the case in the Manitoba (see above).

This paper adds to Canadian and international scholarship on HIV care by highlighting providers’ perspectives on what facilitates HIV care and changes that are needed to improve care uptake and retention, within the particular context of Manitoba’s HIV epidemiology and social issues (i.e., houselessness, injection drug use). Drawing upon interviews with HIV service providers, we examine two questions: (1) What facilitates PLHIV access to and engagement in HIV care? and (2) What changes are needed to improve access and engagement in HIV care? Using a socio-ecological model, we examine the range of facilitators and recommendation at the individual, interpersonal, organizational, and structural level, demonstrating the need for multi-level change and highlighting service providers’ expertise and experience as important knowledge in improving HIV care.

## Methods

The full protocol for this study has been published previously (32); the methods described in this manuscript are abbreviated and specific for the results presented. This study was conducted using participatory action research with extensive community-based participation, and collaboration from interdisciplinary researchers, community organizations, peers, and a diverse Research Advisory Committee.

### Context

Manitoba is located in central Canada, and historically, on ancestral lands of the Anishinaabeg, Anishininewuk, Dakota Oyate, Denesuline and Nehethowuk Nations, and the homeland of the Red River Métis (33–35). Indigenous people are overrepresented in HIV diagnoses in Manitoba (7). Researchers have documented the health disparities between Indigenous and non-Indigenous people in Canada (36–38), emphasizing how past and current structures of racism and discriminiation have resulted in health disparities, socio-economic inequalities, alarming rates of violence (e.g., Missing and Murdered Indigenous Women and Girls) (39), incarceration (40, 41), and disruption to families and communities by continued seizure of Indigenous children by child welfare agencies (42). Further, it has been well-documented that racialized individuals are disproportionately affected by diseases such as HIV and COVID-19 due to social and structural inequalities (43–47). Systemic racism in the Canadian health care system has resulted in disparities in both access (48) and health outcomes for Indigenous peoples (38, 49).

### Setting

This research was conducted with participants in Winnipeg and Brandon, the two largest cities in Manitoba, and Swan River, a small town in west-central Manitoba. Manitoba has a population of almost 1.4 million, with about 900,000 people residing in its capital city Winnipeg (50). HIV care in Manitoba is coordinated through a centralized Manitoba HIV Program. Upon HIV diagnosis, clients are referred to the Program which assigns people to care in one of three clinics: Health Sciences Centre HIV Clinic (Winnipeg), Nine Circles Community Health Centre (Winnipeg), and 7th Street Health Access Centre (Brandon).

### Participants

We used purposive (51) and convenience sampling (52) to recruit a cross section of HIV service providers. The specific characteristics we identified for maximum variation included job roles across the HIV care continuum (see Table 1), geographical location, and length in HIV program. Recruitment included contacting employees at the three Manitoba HIV Program clinics, requesting referrals from other providers, and contacting other health and social service organizations that serve PLHIV and PLHIV who use substances. Snowball sampling was also used, with participants recommending other practitioners. Forty service providers were contacted by email with a short summary of the research project and an invitation letter requesting their participation in an interview.

**Table 1:** Roles of service provider participants.

Role of service provider	Number of participants (*N* = 27)
Pharmacist	1
Health Education Facilitator	1
Community Health Nurse	2
Nurse Clinician	2
Social Worker	2
Public Health Nurse	4
Nurse Practitioner	5
Program Manager or Director	5
Physician/Clinician	5

### Data collection

Virtual semi-structured interviews with service providers were conducted between October 2022 and January 2023; data collected ended once data saturation was reached and there were no new themes discussed in new conversations. Once participants agreed to take part in the study, they were asked to select an interview time using an online scheduler, after which they were emailed the consent form to read and sign before the interview. The consent form was reviewed again before the interview started and to obtain verbal consent. Interviewees were asked to speak about their experiences as a service provider, including their perspectives on institutions, structures, and policies related to HIV care in Manitoba, and what they believed facilitated people’s access to and engagement in care (see Supplementary material 1). Interviews ranged from 45 to 90 min in length, and were audio or video recorded using Zoom or Microsoft Teams. To ensure confidentiality and privacy during virtual data collection, we followed the University of Manitoba’s Guidelines for Virtual Research Involving Participants. The research team offered guidance over email and phone calls in case participants wanted additional information or support related to their virtual participation. Video recordings were deleted after the session for participant confidentiality. The interview guide included 19 questions and was co-developed with our Research Advisory Committee to ensure it captured our research questions (32). The guide was divided into five categories: (1) structural, systemic and organizational barriers to engagement and retention in HIV Care; (2) facilitators to engagement and retention in HIV care; (3) impact of the COVID-19 pandemic on PLHIV, service providers and the health care system; (4) recommendations for policy and programmatic changes to improve HIV care and conditions for service providers delivering care; (5) knowledge mobilization strategies to support education and prevention of HIV and STBBI’s.

### Data analysis

Interview recordings were transcribed using Otter.ai and reviewed for accuracy by two persons. Transcripts were uploaded to NVivo 12 Pro qualitative software, which was then used to perform a thematic analysis of the data (53). Thematic analysis is a method whereby researchers code qualitative data, develop themes, and interpret patterns and concepts, which are then reported using a conceptual framework or model (54). Drawing upon Braun and Clarke’s six step method for thematic analysis (53), we began with three researchers each performing line-by-line coding on a subset of four transcripts, to develop an initial codebook. After discussion, we further refined the codebook which was used to code the remaining transcripts. As coding progressed, the three researchers continued to refine and modify the codes as insights and patterns were identified in the data. Once all transcripts were coded, codes were grouped into themes and reviewed by three researchers for inter-coder reliability and theoretical saturation. Further data analysis was done to group data into the five levels of the social ecological model: (1) Intrapersonal (i.e., individual characteristics and behaviours); (2) Interpersonal (i.e., relationships, support networks); (3) Socio-cultural/Community (i.e., interactions and communications between organization and institutions); (4) Institutional (e.g., healthcare systems, organizations); and (5) Structural (i.e., policy).

Drafts of the manuscript were reviewed by some study respondents to validate (55) that what was recorded and analyzed was in line with their views. We employed the process of member checking and invited three participants to review drafts of the manuscript to validate the researchers’ interpretation of the interview data and analysis. The respondents provided additional edits and comments, which were considered by the researchers and adopted where appropriate. The main change to the data proposed by the participants was to the recommendations where they clarified one of the needs of the Manitoba HIV Program that they felt was not fully captured in the manuscript draft they reviewed.

## Results

Our final sample included 27 participants who held roles ranging from front line providers to program managers (Table 1). Participants self-identified as women (*n* = 20), men (*n* = 5) and non-binary (*n* = 2).

The data are presented in aggregate form in this paper, across participant roles and personal identities (Table 2), drawing on direct quotes and our own analysis.

**Table 2:** Providers’ perspectives on facilitators to HIV Care within the social-ecological model.

Factor Level	Facilitator	Sub theme
Intrapersonal	Non-judgmental care	
	Acknowledging privilege & bias	
Interpersonal	Relationship building	Collaboration with clients
Service Provider	Collaboration with client	Harm reduction approach
		Flexible approaches to client care
		Consistency and follow-up
	Education and knowledge sharing	
	Supportive workplace	Teamwork and staff training
Institutional	Access	Outreach strategies
		Flexible and low barrier care
		Basic needs and social supports
	Safe, non-stigmatizing environments	Accessibility
		Inclusivity and cultural safety
		Harm reduction approach
	Wrap around HIV care	Staff and team roles
	Collaboration with other providers & referrals	
Structural	Improved access to HIV care in rural and remote communities	
	Policies	Infant formula, PrEP coverage

### Intrapersonal

#### Non-judgmental care

Participants across all roles highlighted the importance of intrapersonal facilitators. Specifically, they explained that a non-judgmental, compassionate, and welcoming approach to care is critical to dismantling the shame and stigma that often prevent PLHIV from seeking care in the first place or staying engaged in care. Participants, especially those working in frontline roles where they engage with patients daily (e.g., nurse practitioners), emphasized that talking openly about sex and substance use and asking their clients questions about how they are engaging in these practices in a non-threatening and curious way, destigmatizes these practices and creates a safe environment in which clients feel comfortable sharing some of the more private and sensitive aspects of their life.

*So, talk about it openly and…create a space where that’s it’s okay to talk about. We offer testing counseling, PreP… and even anytime I see a young person, talk about it and try to destigmatize it, even if a young person is coming for physical. Are you sexually active? Okay. STBBI testing is just part of that, right? And not having that stigma attached to it*. (Participant 15)

*When I first see people, for intake and stuff and I know that that they use… I make it very clear that I do not care that they are using because we always encourage people, you know, to live healthy…but also understand that they have been diagnosed, they are not coming here because they are either wanting to get off drugs or whatever else. I’m going to support them, whether they are using, not using, if they are using too much, or if they want to get off the substances*. (Participant 11)

Some providers were grateful because they felt all staff in their organization (e.g., administrative and frontline support staff) were non-judgmental and kind—an important quality given they are the first faces many people see. While their clients may have been treated poorly elsewhere, feeling welcome and accepted in their organization is an atmosphere they can control. As one provider working in an educational capacity commented: “*You may have been the only care provider who was really kind to them that day. And that is so very important”*. (Participant 24).

#### Acknowledging privilege and bias

Several participants recognized the privilege that comes with being a healthcare or social service provider, and the negative connotations or distrust PLHIV might associate with their professions.

*I know that with social work comes a very complicated history as a profession. And so, I really work hard to check myself and realize that I have a lot of privilege and that if I’m sitting, judging someone, it comes from these places of privilege that I experience… I might make a judgement when someone’s coming violently at me or aggressively at me, but I also remind myself that that comes from somewhere, it comes from privilege, it comes from my own experiences, and that the other person sort of displaying those behaviours towards me, it comes from a place too that I have no idea about and I do not know where it comes from for them. I really try to check my judgments, so I am aware of them and can set them aside*. (Participant 2)

Participants recognized that more work is needed beyond simply “*acknowledging*” privilege or bias; they described taking time to reflect on assumptions they themselves may make about clients and how they work through this using an empathetic approach to care delivery.

### Interpersonal

#### Relationship building

Providers, especially those providing direct care to patients (e.g., nurses, educators), explained that a critical part of their role was relationship building: “*a lot of what we do is relationship building.”* Several providers explained the relationships they develop with clients facilitate the trust and safety required for good care. To do this, providers say they collaborate with clients to develop a care plan based on the clients’ priorities and needs; apply a harm reduction approach in their practice; are flexible in how they deliver care; and communicate and follow up consistently with clients. Participants noted that such relationship building can be slow and requires patience and understanding. As one participant explained, even when a particular interaction is not successful in facilitating HIV treatment, any care that addresses even just one health need is important.

*I think inherently in our approach to patients, and one of our priorities, something we are always mindful of, is building trust, [it] is a huge thing, right? Like, HIV is attached to stigma and drug use is attached to stigma, and being gay, or bisexual or whatever, that’s all attached to stigma. …I’m always aware of in my work, my communication style, taking my cues from the patient. And making them comfortable that way*. (Participant 4)

*We try to be that trusting bridge where…I literally will sit with my clients for an hour, an hour and a half, hear their whole story. And whatever they want to tell me, ask questions, really, fully listen, and then be like, how can I help you? These are the things I could offer, “Is this something you’d like to do today?” And where else in the healthcare system do you get to sit with a client for an hour, and just listen, so there’s like this kind of small counseling session that happens. But it’s that trust, it’s that rapport, so then… you are working with them side by side. So, they are much more inclined to actually do that*. (Participant 9)

Similarly, providers across all roles described how collaboration is an important way to respect clients’ agency, providing them with options and resources for their own decision making. They explained that this means asking questions, sharing information and education with clients, and working together to come up with a plan. As participant 6 put it, “*So, the goal is what the patient’s goal is, right?”*

And Participant 11 explained:

*I make sure that it’s very clear that. it’s not really on our terms, like, we work for them… I tell them, I work for you, you decide what you want when you are here. I have to do bloodwork and ask you these questions, but you know, you are pretty much the boss, right? And so, I want them to feel comfortable that they can share and trust that I’m there for them and their health in whichever way they see what health is to them.*

#### Harm reduction

We asked participants to tell us what harm reduction means to them and how they apply it to their work. Almost all providers, regardless of their specific role, spoke of harm reduction as a philosophy that is simply about reducing harm in one’s everyday actions, not just substance use. Consistent with this approach, providers believed it was important to accept that some clients use substances and not demand they change. This mindset, participants explained, fosters connection with clients, facilitates safe conversations about drug use and sexual practices, and helps providers understand their clients’ lives, and offer the appropriate supports, information, and resources:

*How do I support people living with HIV, and who use substances? I have very honest and vulnerable conversations with them. And it is a lot of that de-stigmatization, and I am vulnerable with them. I listen, and I connect with them. Because like I said, the point of every single client interaction is to build trust. So, and it’s having that harm reduction philosophy… treating people with dignity, respect, listening to and working with them*. (Participant 9)

*Harm reduction to me, the main thing is connection and community care… and meeting people where they are at, so giving people the space to be who they are, regardless of who that is. And giving them the space to be honest, not only with themselves, but with us around what substances they are using. How can we support them? What are realistic goals for them?*. (Participant 24)

#### Flexibility

Participants emphasized accessibility, flexibility, and responsiveness as important characteristics of their practice. They told us they use a variety of strategies to keep clients engaged in care, including reminding them about upcoming appointments, following-up about new medications and side-effects, and having predictable clinic schedules. Consistently letting clients know they are available if or when they are ready to accept care is another strategy to keep clients in care.

Making time for clients whenever they show up, providing their email addresses to facilitate direct communication, engaging people’s partners in their care, being available to deliver care in the community and outside typical “working hours,” and rescheduling any missed appointments were additional ways providers sought to demonstrate their respect:

*Just realizing and not holding on too tightly to be like, you [client] need to come at this time. If they drop in late in the day sort of being like, oh, you know what, I’ve actually got to see this other person… but hey, you know what, if you could stick around maybe I could see you for a few minutes. So true flexibility just to help them and meet where they are at*. (Participant 2)

#### Education and knowledge sharing

A significant part of participants’ interactions with clients is focused on sharing education and knowledge about HIV and other STBBIs, HIV treatment options, prevention, and harm reduction strategies. Providers explained that they also share useful skills with clients, such as how to do basic wound care, or how to use drugs safely (e.g., how to use cookers, where is the best place to inject a needle). Participants reported that education is often a repetitive part of client interactions, consistently sharing the same information and knowledge to help their clients absorb information in easily digestible doses:

*So, I feel the way that we frame people to normalize what they are doing, in an incorrect way first, helps people not feel judged, and then provide an alternative or an educational sort of spin on it. And small amounts of information at a time; I always pick one or two things… sometimes they are like, I put glue in my eyes. That’s a bad idea…but then we do not go on about all the other things that they are doing. That’s overwhelming. Because the plan is that I hopefully build enough relationship to follow up with them and slowly help make change. Not all at once*. (Participant 6)

Several providers shared that as part of their routine conversations with newly diagnosed PLHIV includes what Undetectable equals Untransmittable (U=U) means.

*We have a conversation with people about …Undetectable equals Untransmittable, and to talk to the partners to say, “Look, if you if you still want PrEP, absolutely, we can connect you to those resources. But here’s good science to tell you that if your partner is on treatment, and they are undetectable, you will not get HIV from your partner. So, if you still want PrEP, that’s up to you, but you do not necessarily need it”*. (Participant 23)

#### Supportive workplace

Participants identified working alongside other dedicated professionals and being part of a cohesive team as important to their ability to perform their roles well and deliver good care for their clients; indeed, participants, regardless of their specific role, acknowledged the importance of working with and alongside other HIV professionals:

*I do not think a clinic like ours could function the way that it does without all of the people working in it. It’s a big clinic…there’s a lot of people in the clinic and I think they are all indispensable to how the clinic runs…And, so I think that teamwork component is a special part of our clinic*. (Participant 23)

Some providers explained that part of feeling supported in their workplace includes a commitment to staff training, ongoing learning, listening to feedback and acting on it, as well as commitments to anti-racism and decolonizing work, anti-ableism and inclusivity training. One participant noted a willingness to have honest conversations with colleagues was valuable:

*And also in a larger context, again, advocating and calling out staff in a constructive way like, “hey, I do not think you should really say that. Because of this [reason], I think that you should be trying this language instead.” And sometimes that goes well with staff and sometimes it does not, but you have to be honest with each other, to protect the clients…. (Participant 24)*

### Institutional

#### Access

Providers identified several institutional approaches that facilitate access and engagement in HIV care. For example, some providers explained their organizations are becoming more flexible in how care is provided, which they consider crucial to engage clients experiencing houselessness or who use substances. This includes adapting care delivery models away from set appointment times to accommodate “*in the moment*” care, implementing strategies to locate PLHIV not engaged in care, completing as much as possible in one appointment, and trying to keep wait times short.

*We’ve begun to kind of move our structure to reserve same day appointments, within this last year. So, all of our schedules now reflect some capacity to see patients on the same day that we try to protect a couple of slots…a day because we understand that, primarily that there’s a new population of folks with HIV who do not actually do well with or are not well served with an appointment time*. (Participant 26)

Further, some providers noted that virtual appointments are more manageable for some clients because it means they do not need to rearrange their day, take time off work, or travel to appointments. For other clients, offering taxi vouchers or bus tickets helps them get to a clinic. Some providers noted that offering clothing, basic wound care supplies, snacks, or access to a food bank at one Manitoba HIV Program clinic are additional incentives to visit the clinic and also help clients meet some of their basic needs.

Several participants provide health services to clients through mobile or in-community clinics. They find that going to clients makes it easier to deliver HIV care:

*My whole goal is always to be in the community. I was street nursing at the very beginning of syphilis outbreak in 2004. Just roaming the streets helping people on the streets. I also do testing at the bathhouse. And I also try to find people who were previously incarcerated that I need to talk to. We also go to their homes, because you know…having children and childcare, and it’s minus 30 [degrees Celsius], and taking the bus and COVID, all those kinds of things. So, I try to meet people, not just have them come to my office, but I’ll go to where you are at, whether it be at their home or another place and offer their testing and treatment there*. (Participant 21)

In other cases, some providers who work in clinics said they value working with outreach teams to coordinate care or doing their own outreach to clients. Outreach helps providers maintain contact with clients who do not have a fixed address or have limited access to phones or internet:

*And we have shown that having outreach, assertive outreach, going to people works to get people engaged and to actually bring them into care, eventually, transitioning them back to the clinic. (Participant 6)*

#### Safe, non-stigmatizing environments

Participants noted that creating an inviting physical clinic space that responds to clients’ needs as a reflection of non-judgmental, inclusive, and destigmatizing care:

*…I think populations that we have really sought to engage with would be people who use drugs and people living with HIV. So, by giving them representation, for example, our harm reduction services* —i.e.*, our needle distribution or safer crack use kits, safer meth use kits, and condoms*—*are front and centre in the building. So, we do not hide it in a corner. We do not make it something secret, we are very open. You need needles, you need condoms, you come in here for that. We also have posters that represent our values around people living with HIV. We do our best to try to dispel stigma by openly talking about HIV and the treatment available… I guess by being an organization that tries to dispel myths, we hopefully create a safer space for people to know that they are not alone. And that we understand, and that we welcome folks into our spaces happily*. (Participant 18)

Several participants commented that stigma continues to affect PLHIV’s ability to get health care and their lives overall. They spoke of internalized stigma, community stigma, and stigma in health care spaces and strategies to address these forms of stigma:

*Somehow family physicians need to have a growing awareness and comfort with working with folks living with HIV. HIV is a chronic condition, they may not have issues with the chronic condition itself, it’s the stigma around that, because that still does exist in the medical field. And so that has to be addressed and dealt with. Because more and more… they [PLHIV] want to stick with their family physicians. I think…community physicians just need a bit of more training around that and understanding and less judgement. Recognizing their judgments and being able to set those aside. (Participant 2)*

As with their personal commitments, providers described that an organizational commitment to harm reduction entails having staff who are well informed about harm reduction philosophy and practice and will provide non-judgmental care and harm reduction supplies and services alongside HIV care. Providers described their efforts to respect clients’ privacy include having conversations about their health behind closed doors and putting clients into an exam room when they arrive at the clinic instead of having them sit in a waiting room. Others described accessibility as offering “safer” washrooms where clients can spend as much time as they need but are checked on by staff every 10 min for their safety.

Several participants talked about the importance of integrating culturally safer practices with HIV/STBBI services, by having cultural support workers available, language translators, and an organizational commitment to anti-racism and inclusivity.

*And then at the lodge we have drop-in testing days…we have knowledge keepers, and sometimes we are doing cedar baths at the same time. When you talk about meth and stuff like that too, we have had a few times where folks who were substance users…came and sat in a circle or met with a Two Spirit Elder or one of the other knowledge keepers. We drummed. What was beautiful, this one instance, where somebody struggling with substances… came, got fed, was taken care of, was given goodies, and then got to use one of our drums and shared song. And we realized the value of that, because that individual came in and was able to be themselves but share and give back in that space. Especially around cultural and traditional items where oftentimes people…especially those using substances are told or believed that they are not supposed to be around or near or touch. So yeah…making sure there’s the right people around to kind of give that love*. (Participant 5)

Overall, providers told us that culturally safe approaches increase accessibility to HIV care and help to facilitate trust and ensure care is delivered in the way that best supports their clients.

#### Wrap-around HIV care delivery

The Manitoba HIV Program care sites are designed to offer as many services as possible at one site. For example, the two sites in Winnipeg each have a staff team consisting of a pharmacist, social worker, and dietician, in addition to nurse clinicians, nurse practitioners, and HIV physicians. The community site also provides primary care services, health education facilitators, cultural supports, and mental health counselors. In addition, this centre has a harm reduction program located near the entrance, which serves the dual purpose of providing harm reduction supplies and ensuring health education facilitators and nurses for STBBI education and care are available.

*We’ve tried to create very low barrier access for people who use drugs, particularly injection meth in our community, by pairing that service with harm reduction, distribution, needles, meth pipes, condoms, etc. They find that works well for them. We’ve also paired the service with some basic needs. The pitstop gives them water and snacks and mittens when it’s cold and sunscreen when it’s hot. So, by meeting their other needs and increasing access to testing, people have also said that works well for them*. (Participant 18)

The hospital-based site also has a psychiatrist and gynecologist available on HIV clinic days. The third HIV program site in Brandon offers similar services to the community site in Winnipeg, with the addition of a tax specialist and laundry machines.

Many participants described the benefits of a wraparound HIV care model and providing as many services in one location as possible serves as a *“one-stop shop”* for PLHIV and may also reduce the frustration from having to navigate services independently.

…*where I work with the Manitoba HIV Program, it’s a team-based approach. So, I would see my patients at the same time as they might have appointments with a dietitian, the pharmacist, our nurse practitioner, or one of the infectious diseases physicians in one physical space, and there is social work as well. So, the advantage there is the one-stop shop and proximity to the lab, they can do all their HIV labs at the same time*. (Participant 25)

Several participants highlighted the importance of specific team roles such as social workers in facilitating access to other necessary supports for PLHIV clients, such as basic needs assistance (e.g., food, income assistance, housing, transportation), and substance use treatment or mental health services.

*So, they [social workers] would be our experts in where else can you access counseling or others support workers? Our social workers will see, oh, you already have [support]*… *we sort of look at, is it the right fit to access our outreach workers? Or are you already in a program that offers transportation to and from appointments? Or have community supports? And what are other*…*housing strategies that we can engage with? If we are not having success, are there other organizations that could help you? And so, our social workers are really the best that navigate that. See, what are the services you need, and where can you access them if we do not offer them?*. (Participant 14)

Similarly, several nurses described how their roles involve a component of system navigation where they support clients with understanding and obtaining services related to managing their HIV care or other co-morbidities and connect them to other health providers or community resources.

*So, I think of us a lot actually as navigators as well as for patients. And I do think of that as maybe not in a formal service, but something that we do on a regular basis to help navigate and connect patients to whatever they might need*. (Participant 4)

#### Collaboration with other providers and referrals

Participants described how they often rely on collaboration with other health care or social support providers to coordinate client care, either within or outside of their organization. Examples shared ranged from public health nurses connecting clients to HIV program care sites, coordinating referrals, outreach teams who locate disengaged clients, or community organizations who coordinate transportation to appointments.

*We have to partner with those who can. So increasingly, we are collaborating with community support, outreach, and that from different agencies. We’ve now got a meeting…with [several health organizations] where we strategize around how we are going to find some patients and how we are going to get them to engage in care* (Participant 1).

Participant 27 explains how they collaborate with colleagues to facilitate access to other health services:

*The advantage, and I think why HIV programs should always be in the hospital, or part of the HIV program, is that we can get things done. I can call a buddy and get things done for a patient if it’s not HIV, right? If it’s heart or liver, or tests like MRI and CT scan, bone marrow, I just call a colleague, and we can get it done. So, that sort of connection with the hospital is important. … and [we have] a good relationship with the emergency department as well. So, we can get people into … emergency. And we try to keep those relationships fairly positive. Help them, they help us, which is huge*. (Participant 27)

### Structural

There were only few structural facilitators participants identified. Participants reported on two provincial policies that support PLHIV: a program that provides full coverage of infant formula for 1 year in Manitoba for a parent living with HIV, and increased coverage for the cost of Pre-Exposure Prophylaxis (PrEP). While their clients living with HIV do not need PrEP, it is something their partners or people at risk of acquiring HIV may consider as important prevention, as well as people who use drugs.

The other structural facilitator one provider noted was that there is now better access to HIV care in remote and rural areas:

*I’m glad that now people can access HIV care in more diverse ways [as] they used to be limited. I know people that in the ‘90s moved to Winnipeg, because that’s the only place they could get to from reserves [First Nations communities]. Because they can only get HIV care if they moved to Winnipeg at the time. And, ended up in a rough situation, for lots of reasons. I’m really glad that now that’s not the case. But now it’s not like oh, you have HIV, you have to move to Winnipeg*. (Participant 19)

### Recommendations

We asked service providers two questions related to recommendations for improvements to HIV care delivery in Manitoba: (1) *What are your suggestions for policy changes or program enhancements, that would support both people living with HIV and service providers delivering care?;* and *(2) What additional resources are needed to better support people living with HIV?*

As with the care facilitators covered above, we aligned and present the recommendations within the socioecological mode (see Table 3).

**Table 3:** Service provider recommendations.

Factor level	Recommendation	Example
Intrapersonal	HIV and STBBI Education	Share knowledge about HIV with PLHIV, people at risk, general public and providers
	Peer support for PLHIV	Support groups, social programming
Socio-cultural/Community	Address HIV stigma	Education and awareness about HIV in communities and among health care professionals
	Increase spiritually and culturally safe care	Integrate cultural practices such as more cultural support workers, ceremonies such as sharing circles, or translation services for immigrants
		More training and education for health care providers in anti-racism and culturally safe care
Institutional	Access	Adapt care delivery model to increase capacity for in the moment care (i.e., walk-in or drop-in appointments)
		Street nursing and increased outreach-based services
	Prevention	Pair education about HIV and STBBI prevention with harm reduction services—in schools and in training for health care providers
		Enhance capacity for primary care providers to offer PrEP and PEP
	Testing	Increase access to testing and capacity for HIV lab testing outside of Winnipeg
	Treatment	Enhance primary care providers capacity to treat PLHIV
		Bolster support for people who are incarcerated and living with HIV
	Programmatic resources	Increase resources to allow for hiring of additional staff to meet demand for services
		Peer engagement in STBBI care
	Improve collaboration with other providers and organizations	
Structural	Increase availability and accessibility of HIV care options within Winnipeg and outside of major centres	
	Legal and Policy changes	Adapt or abolish HIV non-disclosure law
		Harm reduction policy changes (e.g., decriminalize drugs, safer drug supply, supervised consumption sites)
		Universal HIV medication coverage
	Adaptations to HIV care delivery	Increase funding for HIV care across the province and address jurisdictional issues
		Adapt Manitoba HIV program to a provincially mandated program
		Adapt health information sharing policies and create common shared database
		Allow a mandate for “HIV Treatment as Prevention (TasP)” with the authority to routinely receive HIV viral load test and ART prescription information
		Adapt fee for service model
	Harm reduction strategies	Increase access to harm reduction supplies and services, and substance use treatment options
	Social supports and comprehensive health services	Increase availability and accessibility of no cost mental health services
		Address houselessness and provide safe, affordable housing first

#### Intrapersonal

Participants noted that providing education about HIV, for both people working in health care and the public, would serve to increase awareness about HIV, reduce stigma, and normalize HIV and STBBI testing as part of routine sexual health care.

*[we] definitely need to educate the people in Manitoba that HIV does exist. It crosses all demographics, you know, people who are living on [street with expensive homes], not just people who are living in the downtown core. And that anyone, if you have sex, you could be at risk*. (Participant 10)

Further, participants noted that specifically for primary care providers, additional education about HIV and STBBI’s may increase their understanding of how best to support clients living with HIV and enhance their comfort with providing routine care to PLHIV and PLHIV who use substances.

*…when you go to your doctor’s office, they should be doing the same kinds of things. You know, like ask those questions, what kind of sex are you having? Do you do drugs?*. (Participant 21)

Several participants said they would like to see peer support strategies implemented such as social programs or support groups where PLHIV can build community and offer support to each other.

### Socio-cultural/community level

Providers noted adaptations are needed to enhance their organizational capacity to deliver culturally safe care. Their recommendations include offering clients spiritual and cultural supports, such as incorporating traditional medicines or having an Indigenous Elder available, and hiring translators so clients can attend appointments to discuss their health, without having to bring a family member such as a child to their appointments. Similarly, providers discussed taking steps to address their own biases and “unlearn” and relearn ways of being aligned with reconciliation and anti-racism. Some providers reported their organizations allocate time for staff training, whereas others described seeking out learning opportunities in their own time.

### Institutional

While many aspects of the current wrap-around HIV care delivery model are working well, providers recommended changes to reach more clients, particularly marginalized PLHIV who are not consistently engaged in care. Many providers recommended moving to more flexible care models that meet clients where they are in communities, such as with street-based nursing and mobile outreach clinics. or to offer more flexible drop-in or walk-in appointments at clinics.

*…providing care where people are. I think we need to do that. We pour money into four walls. This is the clinic, that’s where they go, and people find safety. And I understand that people are concerned for their own health too. But we really need to remove ourselves from those four walls and get out to where people are, you know?. It’s difficult to get to appointments. And then when you have other social economic issues, or any of those kinds of things that affect your ability to find care and seek care. We need to break those barriers down and go to where people are*. (Participant 21)

Several recommendations related to HIV treatment specifically, such as improving linkage to care at time of diagnosis, increasing support for PLHIV who were incarcerated to ensure their care remains consistent once they are released, and increasing primary care providers’ (i.e., physician or nurse practitioner) capacity to provide routine treatment for PLHIV, referring to an HIV specialist as needed.

*…what we are working towards is…any primary care provider would have the capacity or opportunity to provide care for people living with HIV, in collaboration and with the support of the Manitoba HIV Program. So, we are building an education framework and a consultation framework, so that clinicians can educate themselves in HIV and also can have access to a specialist on a day-to-day basis from the HIV program, so that we can empower them [primary care providers] to deliver the care*. (Participant 8)

One participant called for more support for PLHIV who are incarcerated, as they explained that it is an opportune time to connect PLHIV to care, however PLHIV can often “*fall through the cracks*” once released from prison, disrupting any care they were receiving.

Related to prevention, providers noted that while PrEP and PEP are available to the general public, there are a limited number of clinics that offer PrEP because of the time required for follow-up testing, and they are referred to one HIV clinic location. Participants also suggested that pairing education about HIV and STBBI prevention with harm reduction services is important for clients and ensuring this is part of providers’ medical education.

*I think medical programs and nursing programs need to address it better. I went to nursing school, I did not learn how to do a genital exam, they skipped the genitals. So, talking about a harm reduction and sex positivity with learning medical practitioners is important*. (Participant 18)

Providers also called for additional resources for HIV care, specifically hiring more staff (i.e., nurses, physicians, social workers) to meet the demand of the number of current PLHIV clients, as well as hiring PLHIV as peer support workers, which would enable both greater involvement of peers in HIV care and offer a type of support and understanding rooted in their lived experiences.

*…have programming that’s led by peers. Like there’s good data that it’s super helpful and just anecdotally…if you have got someone that you could see yourself in to help guide you and has lived experience. I know what you are going through and can help someone newly diagnosed or not even newly diagnosed, just living with HIV and can help navigate some of the difficult times, or situations to connect to care and services that might be helpful*. (Participant 14)

### Structural

Participants all noted the need for change at the structural level, with participants suggesting a range of different recommendations. Overall, we identified five main themes of structural recommendations from provider interviews. First, some recommended increasing the availability of HIV care in rural and remote communities as care is often fragmented and difficult to access due to location and availability of providers. Additionally, several providers called for more options for HIV care within larger cities (i.e., Winnipeg and Brandon), such as by increasing the number of locations that offer STBBI care, increasing the number of centralized locations where people could receive HIV care, and establishing other options for non-urgent after-hours HIV/STBBI care.

Participants identified several key legal and policy changes to address system inefficiencies, including changing policy for HIV medications to be fully funded by the government, adapting or dismantling the HIV non-disclosure law, and for changes to laws and policies around substance use and harm reduction (e.g., decriminalizing drugs, supervised consumption sites). In June 2024, after the interviews were conducted, the provincial government did announce a new policy for HIV medication coverage, making PrEP, ART, and PEP available at zero cost to Manitoba residents.

Several participants spoke about the punitive nature of the HIV non-disclosure law in Canada and the negative consequences for PLHIV. In 2012, the Supreme Court of Canada imposed under criminal law that in some cases PLHIV must disclose they are living with HIV before sexual activity if there is a “realistic possibility of transmission” and can be prosecuted for failure to do so (56). However, the HIV Non-Disclosure law is complex and has been under scrutiny for years, given that it can further criminalize HIV and has been linked to experiences of violence (57). Participant 24 elaborates:

*I do not think that these disclosure laws fully encapsulate what U=U means. And so, I think there needs to be some changes to that. I think that the disclosure laws also do not take into consideration that people have been killed for disclosing their HIV status, because there’s still so much stigma and misinformation around HIV. Like there’s still tons of folks that will get a new HIV diagnosis and think that it’s a death sentence. So, when you have someone who discloses their HIV status to their partner, after sex, the partner often has that misinformation. And if they do not kill the HIV positive person, they can be very, very violent. And I do not think it takes that into account enough as to like how negatively impacted someone could be by that*. (Participant 24)

Participants shared several recommendations for changes to the way HIV care is delivered in the province including increasing overall funding for HIV and STBBI care. Another structural policy change advocated for by several participants was strengthening the ability of the Manitoba HIV Program to safely and proactively coordinate and administer HIV care in the province.

*We are a concept; we are not program. And because without a provincial mandate…that makes it…sort of incapable of advocating for resources in any kind of capacity. If we were entrusted to measure the international 90–90-90 indicators for the Province of Manitoba, well, then we could say, we need an evaluator to do this on a routine basis, but we do not. We do not have an identity. So, now I think the next things that need to come, I relate to defining who we are, what our mandate is. And allowing some of those other program administrative pieces to fall into place*. (Participant 8)

After some respondents reviewed this manuscript, they refined and provided additional insight to a structural recommendation to improve HIV care in Manitoba. Providers envisaged the Manitoba HIV Program having the authority to routinely receive HIV viral load test and ART prescription information—a “Treatment as Prevention” mandate—and institute a provincial database similar to those used in other provinces, such as British Columbia. This would allow the implementation of fail-safe mechanisms to identify gaps along the HIV care continuum and allow the Program to coordinate support for individuals who may require additional resources to be successfully re-engaged in HIV care. Some participants felt that for this to happen, the Manitoba HIV Program may need to transition to be a formal program within the provincial health authority of Shared Health.

At the time of the research, there were no policies that specifically support harm reduction services such as supervised consumption sites or large-scale monitoring of an increasingly toxic drug supply in Manitoba. Substance use treatment options, particularly for people who use methamphetamine, are sparse. Given that injection methamphetamine use is one of the main drivers of new HIV diagnoses in Manitoba in recent years (7, 11), participants in our study highlighted the importance of both policy changes (e.g., safer drug supply, decriminalize drugs, supervised consumption sites), and increased availability of substance use treatment options as necessary for reducing transmission of HIV in the community, and providing lifesaving support for PLHIV who use substances.

*As they [government] need to figure out what their next step is around substances in general, they need to decriminalize drugs and find a different way to navigate that, because it’s what’s driving the demand on safe supply. And trying to put something like this on the backs of physicians in a country that can barely sustain the amount of physicians that we do not have what we need already. Physicians should not be navigating safe supply; they need to legalize substances and find other health and social service mechanisms to create safe environments for people who use drugs and not put this on the traditional medical system. So, those kinds of substance-related, drug related laws, are probably the place to focus on*. (Participant 7)

Most providers discussed how increasing socioeconomic disparity and their clients’ inability to meet their basic needs is a major deterrent to engagement in care; this includes providers acting in frontline care roles and those in director/managerial roles. Participant 7 noted, “*what’s missing is a social determinants strategy as part of health care.”* Accordingly, addressing housing needs, transportation accessibility, and income disparity are necessary steps toward facilitating people’s medical care:

*…if we do not address the social determinants of health, people cannot address their medical needs they really cannot, because it’s so far down the priority list. You know, having a safe, warm place to sleep and having food is going to be people’s survival. That’s what they need*. (Participant 10)

Participants recommended implementing solutions such as housing first strategies, guaranteed basic income, low-cost or no cost province wide public transportation, and ensuring everyone can access enough food.

## Discussion

In this paper, we examined service providers’ perspectives on facilitators to HIV care and their recommendations for improvement, based on their occupational experiences and perceptions. By categorizing facilitators according to the social-ecological model, we demonstrate how factors at different levels interact to support access to and engagement in HIV care. Given providers’ roles navigating client relationships and health care system processes, they offer unique insights at different levels of the model, demonstrating that factors at different levels facilitate care Additionally, using this model to categorize provider recommendations highlights where targeted interventions are needed to better meet the health needs of PLHIV in Manitoba.

As with other studies (3, 17, 58) reporting on facilitators to engagement in HIV care, our findings demonstrate the crucial role of the client-provider relationship. To develop trusting relationships, providers must demonstrate a high level of patience, understanding, and empathy to deliver safe and inclusive person-centered care. Participants described their interactions with clients as collaborative and focused on providing information and education, indicating their actions are respectful of their clients’ agency and serve to empower them to make decisions regarding their health, rather than be told what they “should do.” Our findings align with existing research with PLHIV that reported that providers who take time to listen to their needs, respond to questions, and provide information in accessible ways, make them feel more comfortable and like a ‘partner’ in their own care (17), especially for clients who face multiple barriers and disadvantages (30). Providers’ perspectives further align with PLHIV’s perceptions in Manitoba about how to best facilitate engagement in care as PLHIV in our larger study, too, many of whom used substances, noted the critical importance of supportive and empathic relationships (59). Our data highlight the importance of wedding HIV clinic care with harm reduction, both as a philosophy guiding HIV care delivery approaches, and in terms of tangible supports, such as providing free drug use supplies (30, 31). Offering these services alongside HIV care has proven invaluable as reported by providers in our study and in the literature (20, 60). For example, a study by Scaramutti et al. (20) that delivered a peer-led telehealth harm reduction intervention for PLHIV who inject drugs found that participants reported struggling with ART adherence due to distrust of the medical system and mistreatment at medical appointments, fear of judgement and social stigma. Study participants reported that harm reduction services paired with HIV treatment, as well as non-judgmental attitudes and empathetic providers, were important factors supporting their engagement in care (20). Similarly, Collins et al. and Campbell et al.’s research emphasize the importance of HIV care within a broader framework of harm reduction supports.

Our findings show that the way organizations operate can facilitate safe care for PLHIV, particularly through the attitudes and behaviours of staff using a harm reduction approach in their care delivery, and by offering wrap-around services in welcoming and inclusive physical spaces. Similarly, Yehia et al. reported on facilitators from the perspective of PLHIV who noted organizational supports, such as receiving several services in one space and positive experiences with staff were important factors that supported their engagement in care (3). Their findings support the importance of intrapersonal factors (i.e., behaviours and attitudes of providers) as well as intuitional level efforts to create safe, welcoming, non-stigmatizing environments that support consistent engagement in care.

As providers in our study and other researchers have noted, in Canada, there is much work to be done to address systemic racism and discrimination facing Indigenous peoples and to increase cultural safety within health care settings (49, 61–63). Providers offered recommendations about how to shift their personal and organizational practices to enhance cultural safety for their clients, such as by incorporating more traditional and spiritual care as part of clinic care, having translators available to break down language barriers, and committing to their own personal (and in some cases organizational) work to address bias and challenge assumptions.

Providers suggested a number of recommendations for structural change. When participants shared their perspectives about what facilitates HIV care right now, they focused on what is in their control, such as emphasizing the importance of intrapersonal facilitators (e.g., relationship building). Indeed, within the socio-ecological model, providers emphasized facilitators at the intrapersonal and interpersonal level as these are more within the providers’ control, reflecting their attitudes towards their work and their patients, regardless of system and organizational constraints. Often it was hard for participants to comment on what was currently “working well” from an organizational or systemic perspective, perhaps in part because these are the areas where service providers experience the most challenges to enhancing care. However, when it comes to recommended changes, our findings demonstrate the need for structural change (e.g., expanding HIV care). Thus, policymakers and service providers should protect and amplify facilitators that are currently working well (e.g., positive relationship building), while at the same time working to eliminate barriers at all levels (i.e., structural, intuitional, intrapersonal, interpersonal). For example, a better resourced health care system and funding for HIV care would make it easier for providers to focus on the aspects of their roles that they see as the priority, such as building trust and relationship building, without constantly having to negotiate and operate under stress in resource strained environments.

### Study limitations

While the sampling techniques we used are methodologically sound, there are other key informants, such as those who work in rural and remote locations and with people who are incarcerated, who may yield additional key insights. Similarly, our sampling technique might have missed those service providers who are in more resource constrained roles to participate in a research study. To address these limitations, we relied on the guidance of our Research Advisory Committee, who included people across different health and social settings, to identify potential programs for recruitment and ensure representation across the cascade of HIV care. As most of the providers in this study resided in the two largest cities in Manitoba, Winnipeg and Brandon, their perspectives are based on experiences in urban centres. While our findings may not be generalizable, they may be relevant in other locations that are experiencing a surge of new HIV diagnoses among people experiencing social marginalization and people who use injection drugs.

## Conclusion

Our findings provide evidence for the importance of including service providers’ perspectives in studies that examine HIV care delivery, as their insights can complement the experiences and perspectives of PLHIV. Service providers, by the very nature of their roles, are facilitators, and an important link between PLHIV receiving lifesaving treatment or not. Thus, how they deliver care can have a significant impact on PLHIV’s health and wellbeing. Systemic gaps, policy inefficiencies, and resource constraints continue to place an unfair burden on providers where they are not always able to deliver the care they want or know their clients need. We call on decision makers to seek input from front line providers and implement their recommendations to improve access to care and how PLHIV experience such care. Specifically, our findings suggest expanding comprehensive HIV care to smaller and more remote communities, wedding HIV care with substance use and harm reduction treatment and approaches, and providing targeted supports and interventions specifically for PLHIV who are socially marginalized, use drugs, and have a history of incarceration.

## Data Availability

The datasets presented in this article are not readily available because individual qualitative participant data will not be available since it contains potential and sensible identifiable information. Data dictionaries, qualitative codebooks, and quantitative data will be available upon request on a case-by-case basis. Requests to access the datasets should be directed to zulma.rueda@umanitoba.ca.
