# Supplementary material for: Service providers’ perspectives on facilitators and recommendations for improving HIV care in Manitoba, Canada
Source: Front Public Health. 2025 Jul 9;13:1585604. doi: 10.3389/fpubh.2025.1585604 (PMC12312654; doi:10.3389/fpubh.2025.1585604)
Supplement: Supplementary file 1 [file Data_Sheet_1.pdf]

Service Provider Interview Question Guide

To be completed by facilitator	*Facilitator Name*		
Participant ID:			
Participating Organization:			
Location:			
Interview date:			
Interview time:	Start	End	Duration
Confirmation of consent signed:			

Thank you for agreeing to speak with me today. My name is [INSERT] and my pronouns are INSERT]. My role with this project is as a [Research Assistant, Research Associate].

Before we start the formal interview questions, I would like to remind you that we are here to speak about your experiences as a service provider [clinic program providers and/or social service employee] to provide additional perspectives on institutions, structures and policies related to HIV Care in Manitoba. The interview should take approximately 45 to 60 minutes.

Everything you share with me will be kept confidential. I also want to remind you that that our conversation will be recorded so your words are captured as accurately as possible, but the recording will be kept securely and destroyed once the study is over.

You have been asked to participate in this interview as you:

1) *Are a clinic service provider or social service employee who works with and/or provides services to people living with HIV or other STBBI's in Manitoba [and/or people who inject drugs]*

Does this statement apply to you?
Yes
No

[If no, relay that they are not eligible to participate in the study. Thank them for their time, and end interview].

For the purpose of our discussion, STBBI Services may include:

- *HIV testing / care*
- *Hepatitis C testing / care*
- *Syphilis testing / care*
- *Other sexually transmitted infection (STI) testing /care*
- *Obtaining safe sex products (condoms, dental dams, pre/post exposure prophylaxis*
- *Information about STBBI testing/care*
- *Counselling related to STBBIs*
- *Community services or supports related to STBBIs*

Do you have any questions for me before we begin?

1. **Could you please describe your role?**
    a. PROBES
        i. Type of organization (Clinic, CHC, Community Organization)
        ii. Position Title
        iii. Main job duties
        iv. Schedule

2. **What services does your organization provide?**
    a. PROBES
        i. Women's health, mental health, addictions?

3. **Please tell me about what services are working well for your clients/patients living with HIV?**
    a. PROBES
        i. Specific supports or programs
        ii. Accessibility
        iii. Campaigns
        iv. New services to deal with COVID-19

4. **What barriers to accessing or remaining in care have you identified for people living with HIV?**
    a. PROBES
        i. Attitudes to some clients
        ii. Limited hours or sites
        iii. Shortages of supplies or staff
        iv. Person's gender identity
        v. Stigma towards infectious diseases, substance use, race, etc.

5. **What barriers have you identified for PLHIV who are actively using substances that may prevent them from accessing care?**

a. PROBES
   i. Attitudes of staff
   ii. Administrative regulations
   iii. Stigma associated with substance use, race, gender
   iv. Behaviour challenges related to substance use

6. **What major changes have you noticed in your clients/patients due to the COVID-19 pandemic?**
   a. PROBES
      i. Increased drug use
      ii. Increased experiences of violence
      iii. Mental health challenges
      iv. Housing instability
      v. Income instability
      vi. Incarceration
      vii. Increase in sex work
      viii. Suicidal ideation
      ix. Anything else?

7. **What are some examples of success factors that you have seen clients employ during the COVID-19 pandemic to ensure they continue to get the care they need?**

8. **How has the COVID-19 pandemic affected the services in your organization?**
   a. PROBES
      i. Changes of hours
      ii. Not enough staff to meet client needs
      iii. Virtual platform challenges
      iv. Increased needs of clients related to drug use
      v. Strain on health care system & referral services
      vi. Impact on staff to deliver services
         1. Burnout
         2. Getting sick themselves
         3. Not enough training for new staff

9. **How has the COVID-19 pandemic affected you personally as a service provider?**
   a. PROBES
      I. Mental health challenges
      II. Burnout
      III. Secondary trauma
      IV. Increased work load
      V. Covid-19 reinfections

10. **How do you support people whose needs fall outside of the services you offer?**
    a. PROBE

        i. Collaborate with other health providers or community agencies to connect people to these services
        ii. Relationships with other providers in the community

11. **What strategies do you currently use to support people living with HIV who use substances?**
    a. PROBES
        i. What additional training, resources, education do you need?

12. **What is your understanding of harm reduction and how do you apply this in your work?**
    a. PROBES
        i. Harm reduction- knowledge, attitudes
        ii. What else do you want to learn about harm reduction?

13. **What strategies do you use to support your clients with prevention of STBBI's?**
    a. PROBES:
        i. PrEP, STBBI testing, counselling/mental health support
        ii. Education/sharing knowledge about STBBI's- how they are transmitted, how are the treated?

14. **What do you personally do or what does your organization do to create a safe space/environment for clients?**
    a. PROBES
        i. Environment- accessibility, meeting clients where they are at, safety
        ii. Training- anti-racism, anti-oppression
        iii. Policies- sexual harassment, violence, covid safety, harm reduction
        iv. Practices- cultural safety, trauma informed care

15. **Can you tell me what you think about the policies in Manitoba related to HIV?**
    a. PROBES
        i. Specific policies working against ability to get, receive or remain engaged in care
        ii. Policies currently working well to support PLHIV
        iii. Policies you would create to support you in your work as a service provider

16. **What are your suggestions for policy change or program enhancements- that would both support PLHIV and service providers delivering care?**
    a. PROBES
        i. Additional options for care, additional services
        ii. Referrals to other services
        iii. Bundling services in one appointment or location (wrap around care)

17. **What additional resources are needed to better support people living with HIV in Manitoba?**

a. **PROBES**
    i. Prevention- i.e. PREP
    ii. Additional options for care
    iii. Knowledge about STBBI’s- how they are transmitted, how are the treated?

18. **What do you think is the best way [how do you want] to learn about prevention and treatment of STBBI’s and harm reduction practices?**

19. We are asking everyone involved in this study to share their gender. If you are comfortable, can you please tell us how you identify?

That is the end of our interview today. *Before we go, I wanted to know if you wanted to share anything else with me? Or if you think we should have asked you about something else?*

Thank you very much for speaking with me. *We really appreciate your time and efforts and the valuable information you have shared with us today.*
